# Supplementary material for: Resilience of mobility network to dynamic population response across COVID-19 interventions: Evidences from Chile
Source: PLoS Comput Biol. 2025 Feb 20;21(2):e1012802. doi: 10.1371/journal.pcbi.1012802 (PMC11870358; doi:10.1371/journal.pcbi.1012802)
Supplement: Attachment — Submitted filename: response_to_reviewers.pdf [file pcbi.1012802.s001.pdf]

Response to reviewers

We sincerely thank all reviewers for their insightful feedback and valuable suggestions, which have enriched our manuscript and strengthened our findings. Below, we provide a point-by-point response to each of the reviewers' concerns, supplemented by additional analyses, figures, and tables appended to the Supplementary Material and referenced in the main text. We have revised the main text to address all concerns, and we hope that the updated manuscript now meets the criteria for acceptance.

Reviewer 1:

1. *<<One of the arguments used in the manuscript to support the resilience of the mobility network is that hotspots classification in terms of outbound mobility barely changes across the multiple waves of the disease. Nonetheless, in my opinion, raw outbound flows are not proper indicators to quantify such resilience as they are influenced by the differences in population among the different cities. Such differences could potentially turn population centers into mobility hotspots, regardless of how mobility was affected by NPIs. Hence, I think that computing hotspots computed according to per-capita out-strength are more suitable to showcase this consistence while removing the population bias.>>*

We thank the reviewer for raising concerns about this important point that needs to be better explained. Our primary goal is to evaluate the variations in the mobility network during the unfolding of the epidemic. The absolute mobility flows directly impact these variations, hence the rationale of our analysis. In contrast, out-strength per-capita does not directly impact the network's structural load and it can be used to investigate behavioral aspects of mobility, rather than its structural resilience.
The reviewer is however right in underlying the importance of investigating relative mobility changes. In the first version of the manuscript, we partially carried out this analysis in the *Results* subsection *Mobility response to interventions in Chile*, in which we investigate the correlates explaining mobility change of first and second wave. To account for the reviewer's suggestion, we further expanded the analysis on per-capita flow.

Despite Chile exhibiting high population heterogeneities among municipalities, we observe that the hotspot rank across the waves is not constant. **Figure 2** shows the Jaccard similarity index between the hotspot sets of different waves. This score is never close to 1, implying that the network hotspots are not driven by population heterogeneity, which is constant during the epidemic, hence the metric allows for a certain degree of variation. This behavior is in good agreement with previous studies that examined mobility hotspots stability (see, for instance, Aguilar, Javier, et al. "Impact of urban structure on infectious disease spreading." *Scientific reports* 12.1 (2022): 3816.) during 2020 NPIs in US

cities. In the new **Supplementary Figure 3** we further show that outflows per-capita are also very similar across the three periods, with changes in hotspots ranks comparable to those obtained with the total outflows (outstrength).

We thank the reviewer for suggesting this analysis since it confirms that municipalities with high trips per-capita behaved similarly across the three periods, highlighting the significance of comunas' per-capita out-strength we obtained in the regression models. In the main text, we rephrased and clarified part of the *Results* Section to better stress and clarify how the hotspots analysis strongly relates to the network structural aspect and why such methodological choice was made. We also added the new hotspots analysis suggested by the reviewer with per-capita outstrength to the Supplementary Information Section *Alternative definition of mobility hotspots*, **Supplementary Figure 3**.

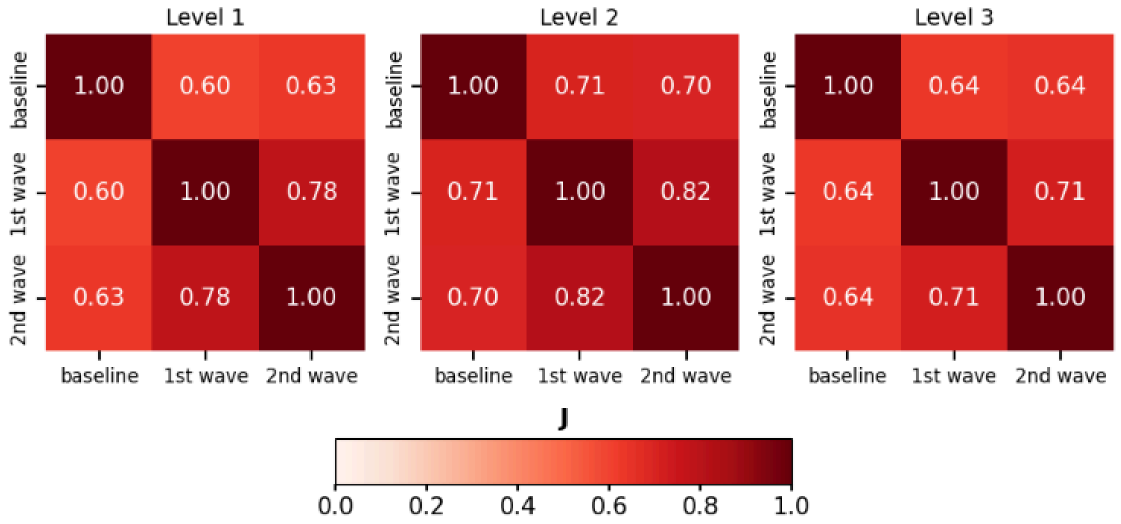

Supplementary Figure 3: Resilience of per-capita mobility hotspots across epidemic waves in Chile. Heatmaps showing the Jaccard similarity J for three hotspot levels across the three periods, i.e., baseline, first, and second wave. Level increases from left to right, with Level 1 encoding comunas with the highest mobility flows per-capita.

2. *<<Another important issue concerns the epidemic simulations carried out to support the similarity of epidemic trajectories corresponding to the mobility networks observed across multiple waves. The authors assume that the force of infection between two nodes just depends on the probabilities of travelling between these two nodes but not on the total number of individuals moving from each node. Neglecting the overall reduction in mobility of the different nodes could allow isolated nodes in the mobility network to propagate the disease across the population, therefore producing quite unrealistic epidemic trajectories. To solve this issue, I would encourage the authors to introduce the overall reduction in mobility of the different municipalities in their force of infection; their findings will hold as long as such reduction is roughly homogeneous across them.>>*

We thank the reviewer for this constructive suggestion. As correctly summarized by the reviewer, the traveling probabilities between the origin and destinations originally employed in our models are normalized by the total mobility outgoing from the origin. This is a standard parameterization, used in metapopulation models. This approximation is based on the assumption that the total outflow measured on mobile phone data is a good proxy for the total population. However, we agree that this approach best fits business-as-usual scenarios, i.e. cases without interventions.

When comparing intervention scenarios, reductions could be underestimated since both the numerator and denominator account for mobility reductions. To investigate this effect, we run additional simulations alongside the original ones. We defined the traveling probabilities as the actual mobility between origin and destinations divided by the total resident population of origin. In this way, the denominator is not affected by mobility reductions and the differences between the 1st and 2nd waves are better captured. We formally define the transition probability as:

$$p'_{ij,P} = \frac{\overline{M}_{ij,P}}{Pop_i},$$

where Pop_i encodes the resident population of each comuna *i* and *P* the period that can be either baseline, wave 1 or wave 2. In the new **Supplementary Figure 4** we show the result of our simulations using three different transmissibility levels, as a sensitivity test. While mobility reductions are not homogeneous across comunas, as already shown in the regression analyses in the main paper, we can appreciate how the hierarchy of invasion is overall well preserved between the two waves. This can be explained by considering the spatial invasion process as a tree of invasion, where the seeding events occur hierarchically from the top (the source area) and the following invaded areas. The denominators, being the total outflows or resident population, only shape the force of infection intensity, without altering the rank of outgoing mobility flows in each comuna, encoded by the numerators. As a second effect, in most low and middle size comunas the population at the denominator is higher than the total outflow for each comuna, hence resulting in a less intense force of infection, and hence a noisier invasion dynamic. However, the heterogeneities of connections between comunas, as already highlighted in previous works on the air transport network (Colizza et al. "The role of the airline transportation network in the prediction and predictability of global epidemics." Proceedings of the National Academy of Sciences 103.7 (2006): 2015-2020), ensure preferential pathways of diffusion, whose rank is hard to disrupt even by heterogeneous mobility reductions, especially for areas most connected with the source. As a result, the invasion times of an epidemic on a heterogeneous network are mostly unaltered by interventions, but only delayed, as previously observed at wider scales (Bajardi, Paolo, et al. "Human mobility networks, travel restrictions, and the global spread of 2009 H1N1 pandemic." PloS one 6.1 (2011): e16591). We have added these considerations and results to the Supplementary Material Section *Alternative parametrization of the invasion model* and referenced them in the Results section ‘Spatial invasion modeling’.

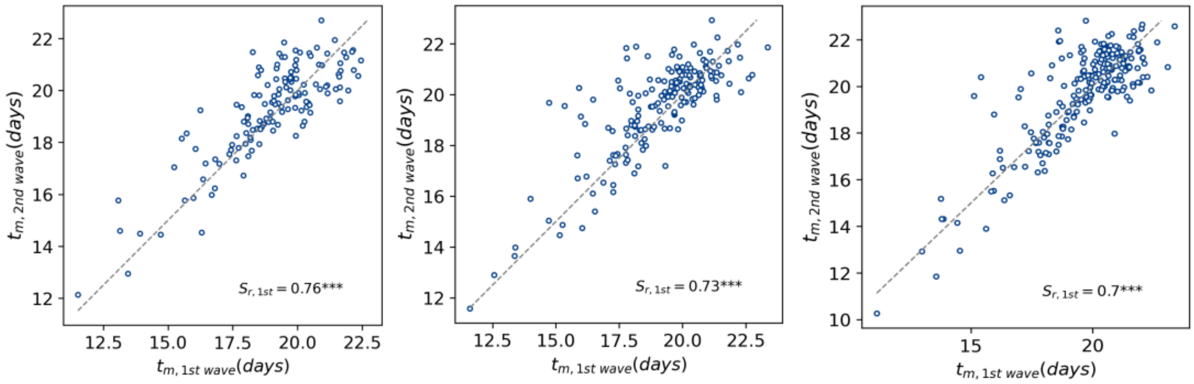

Supplementary Figure 4: Sensitivity analysis for traveling probabilities p_ij and transmissibility β. (a) Average arrival times t_m at municipalities resulted from the epidemic model run on the first and second wave static networks with β = 0.6. **(b)** with β = 0.7 **(c)** with β = 0.8. The dashed gray line is the identity diagonal, and S_r is Spearman's correlation coefficient computed between the two modeled average arrival times.

3. *<<Figure 1: It is not clear to me what the color code means. The authors should better explain it in the caption.>>*

Following the reviewer's suggestion, we improved the figure by discretizing the colorbar with4 distinctive colors and we rephrased the caption to better explain the legend of the heatmap. See below the new **Figure 1** and the new caption.

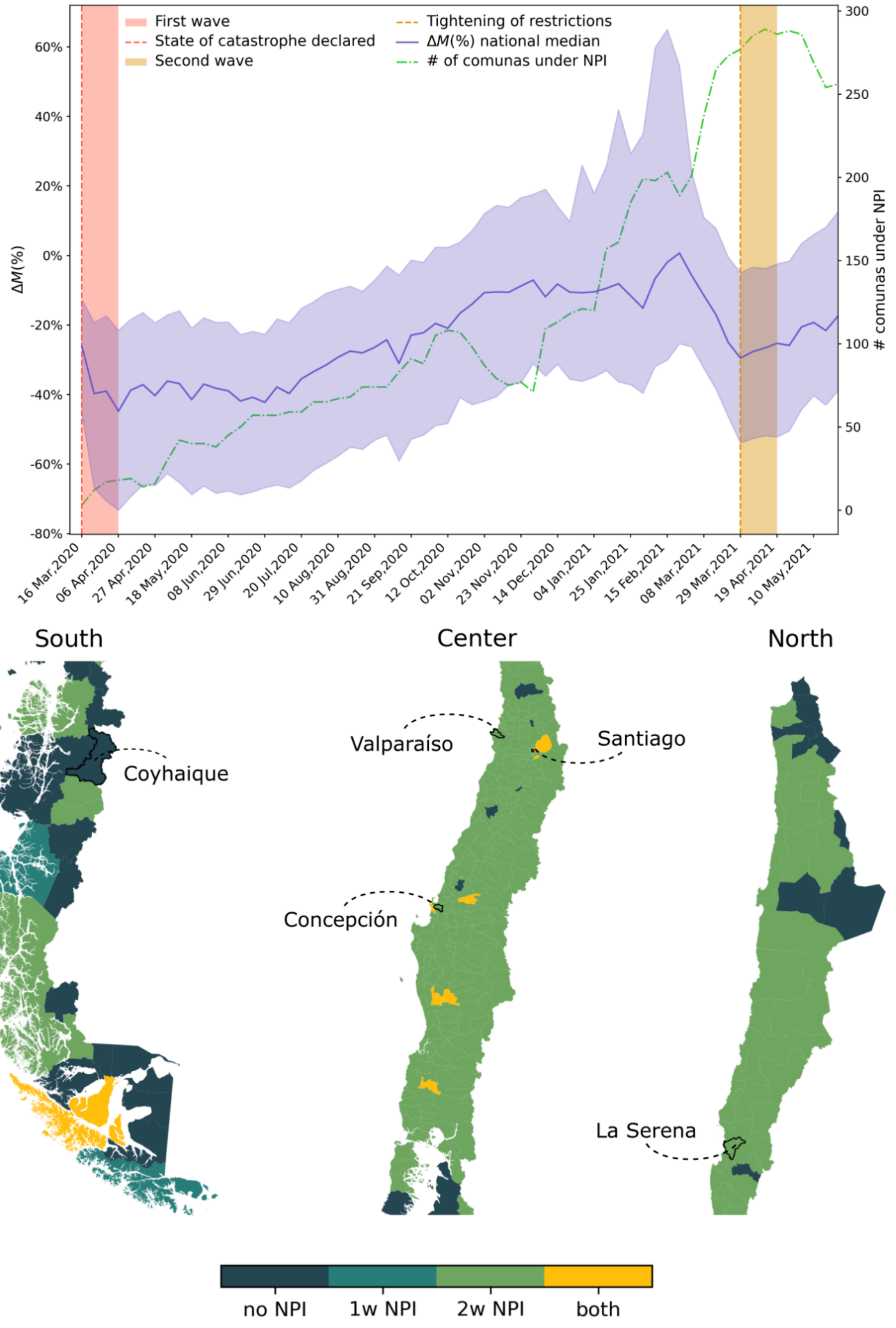

Figure 1. The first and second wave in Chile. On top: weekly median mobility change with respect to the baseline (purple solid line) and respective 95% interval across municipalities (purple shaded area). The dashed-dotted green curve represents the number of municipalities under restrictions, while the vertical areas correspond to the first (red) and second (yellow) waves. On the bottom, the map of three macro-regions of Chile shows municipalities under restrictions. The color code represents municipalities according to the NPIs they experienced. We make the distinction for adoption only in the first wave (light blue, 1w NPI), only in the second wave (green 2w NPI), in both waves (yellow, both) and neither (dark blue, no NPI).

4. *<<Figure 3: I do not see the point of including Panels d and f as the spatio-temporal information of the similarity of epidemic trajectories is already shown by panels c and e. In addition, the authors* ***should explain in the caption that the static networks corresponds to the average mobility networks observed in each period.>>***

The plots d and f of Figure 3 indeed only provide a different representation from the one proposed in panels c and e, as correctly stated by the reviewer. We agree that this information is redundant and we thus updated Figure 3 and its captions.

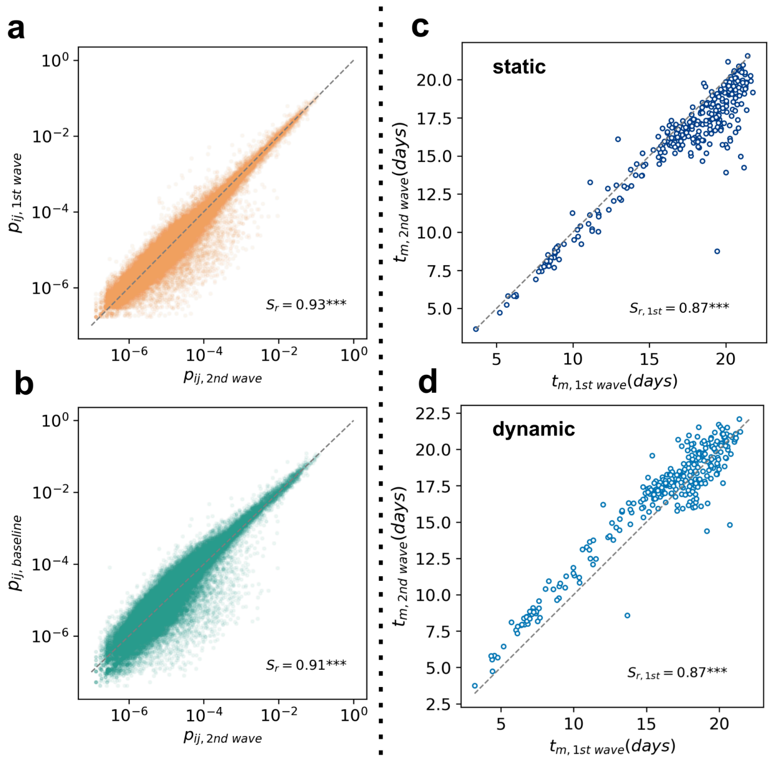

Figure 3. Epidemic modeling on Chile's two waves mobility networks. (a) Traveling probabilities pij computed from the first wave network against those computed on the second wave network. **(b)** Traveling probabilities pij computed from the second wave network against those computed on the baseline network. The gray dashed line is the identity diagonal. The quantity Sr denotes Spearman's correlation coefficient computed on traveling probabilities. Average arrival times t_m at municipalities resulted from the epidemic model run on **(c)** the first and second wave static networks describing the average mobility flows observed in each period, and **(d)** on the dynamic networks. The dashed gray line is the line x = y, and Sr is Spearman's correlation coefficient computed between the two modeled average arrival times.

Reviewer 2:

1. *<<[...]What strongly strikes me, though, is the utter lack of historical analysis. This is only the most recent of many epidemics in the country over the past centuries, from colonial times through the present. It is impossible to derive meaningful results without historical analysis. Simplistically using data from a single even without understanding it as part of a long-term historical process results in very little understanding of the basic processes involved. It will require major historical research to generate that sort of meaningful understanding of the epidemic.>>*

We agree with the reviewer that a deep and broad understanding of the epidemic must be put into an equally broad historical and cultural context. In this perspective, the analysis and experience from previous epidemics would certainly generate useful knowledge.
Nonetheless, while Covid-19 was not the first pandemic to hit Chile, it constituted a unicum in terms of the amount and quality of data generated. These data are at the basis of our analysis and they are lacking in a longer historical horizon.
More precisely, previous pandemics, such as H1N1 set the basis for spatial transmission analyses and epidemic models at the global scale using network science and mobility modeling (see Balcan, Duygu, et al. "Modeling the spatial spread of infectious diseases: The GLobal Epidemic and Mobility computational model." *Journal of computational science* 1.3 (2010): 132-145; and Dirk Brockmann, Dirk Helbing,The Hidden Geometry of Complex, Network-Driven Contagion Phenomena . *Science* **342**, 1337-1342(2013). DOI:10.1126/science.1245200 as examples). Previous studies, such as in the context of the SARS outbreak of 2003, used network science methods to understand the level of risk of importation of the virus at country level and the potential impact of health authorities travel advisories. More recent outbreaks, like the Ebola outbreak of 2014 allowed for higher-resolution insights using mobile phone data to proxy mobility flows across African countries. However, the 2020 pandemic has been the first pandemic in which we have been capable of diving into epidemic dynamics at the municipality resolution to understand the complexity of spatial transmission of diseases. In conclusion, we understand the reviewer's point of view and respect the reviewer's perspective, but from our quantitative point of view, we believe a historical contextualization of quantitative and data-driven analyses falls beyond the context of our study.

Reviewer 3:

1.<<*First, while your models predict certain routes and municipalities as critical in terms of mobility, it is not clear whether these predictions were compared with real-world outcomes during the COVID-19 pandemic in Chile. Specifically, were the routes identified by the model as the most critical also the ones most affected by the pandemic? And were the municipalities connected by these routes among those that experienced the greatest*

impacts? A comparison of these predictions with actual epidemiological data could strengthen the robustness of your findings and provide further confidence in the applicability of your model. I would appreciate it if you could clarify whether such a comparison was considered or whether there are plans to explore this in future work.>>

We appreciate the reviewer's question on the relation between the most resilient routes and the observed epidemic impact. Answering such questions is challenging with the data employed in this study, and more reliable data sources would be needed to provide a robust and accurate assessment. In this work, we employed cases and deaths. In the first case, they are strongly biased as residents tended to perform PCR tests or visit a hospital in municipalities that were not necessarily their own. Various random programs of active search for cases, and changes in the definition of cases also proved counting cases very difficult. Deaths count, on the other hand, constitutes sparser information since it encodes orders of magnitude lower values when working at the municipality level.

To answer the reviewer's first question: "***were the routes identified by the model as the most critical also the ones most affected by the pandemic?***" other studies employed more reliable data sources coming from genomic surveillance and analyzed the most important routes of transmission of SARS-CoV-2 variants in Chile, see Gutierrez et al. Routes of importation and spatial dynamics of SARS-CoV-2 variants during localised interventions in Chile, *PNAS Nexus*, 2024; page 483, https://doi.org/10.1093/pnasnexus/pgae483. In our opinion, these types of studies provide a better and more reliable answer to the reviewer's question than what we could achieve with case counts, because of the biases intrinsic to case data generation and reporting. Previous studies have analyzed the impact of mobility with the same mobile phone data source on local epidemics, see Gozzi et al. "Estimating the effect of social inequalities on the mitigation of COVID-19 across communities in Santiago de Chile." *Nature communications* 12.1 (2021): 2429.

Regarding the second question "***were the municipalities connected by these routes among those that experienced the greatest impacts?";*** The brief answer is "we expect that to be so", but we cannot prove it with the data we have. The most resilient routes, which were also the prevalent outbound routes for *comunas* as shown in **Fig.3a,b**, have potentially played a critical role in the spatial transmission of SARS-CoV-2 across Chile. However, the fact that transmission effectively occurred through these routes is hard to prove with the data we have due to the high complexity of the factors intervening in the spatial transmission, e.g. social distancing measures, multi-seeding (i.e. areas importing cases from multiple epidemic sources), internal mixing heterogeneity, etc. Assuming that we could still see the effect of the main mobility routes resilience on epidemic impact and assuming that "impact" can be measured as the local number of active cases, the destination of the most resilient routes of **Fig.3b** should exhibit higher infections than others. **Fig.3b** shows values for all mobility origins in Chile, so for the sake of visualization, we reproduce the same plot for three major cities of the country.

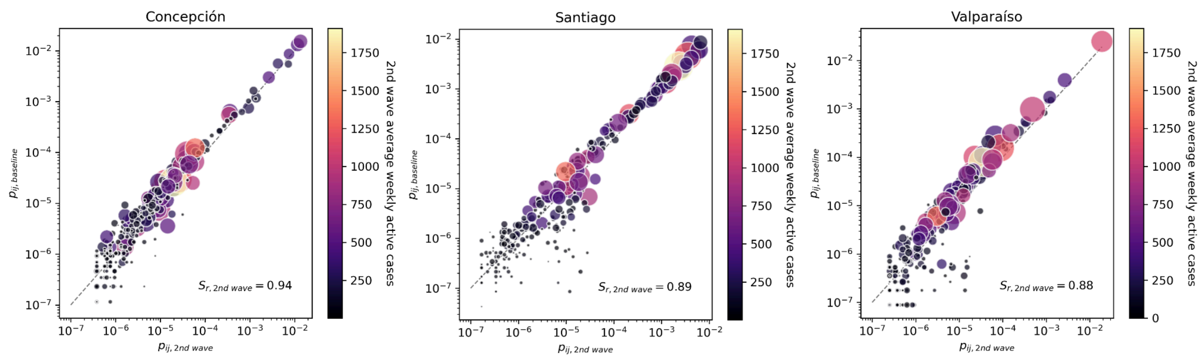

Supplementary Figure 5. Insight on Figure 3b for three major cities. On the y-axis the traveling probability from three major municipalities *i* to *j* in the baseline period vs the second wave period on the x-axis. Circles correspond to destination municipalities, circle thickness encodes population size, and color code represents the average active cases registered in the 2nd wave period weeks. S_r is Spearman's correlation coefficient.

In **Supplementary Figure 5** we see that the secondary routes outbound from Concepción, Santiago, and Valparaíso, in the bottom left corner of the plots, head exclusively towards low-populated and low-transmission areas. However, the most resilient and prevalent routes outbound from the three cities (in the top right corner) include a mixture of high, medium, and low transmission destinations, meaning that multiple factors beyond mobility have affected their local epidemics. Moreover, most hit areas typically correspond to most populated areas, meaning that incidence would not exhibit such a clear signal. In conclusion, there are hints that the least resilient and secondary routes were headed towards low transmission areas, but the opposite effect is harder to prove from such a simple comparison. Hence, we cannot draw definitive conclusions on the effect of the prevalent routes of mobility on local epidemics.

We apologize that in the text we were not clear enough in stating that we are exclusively looking at the structural changes in the mobility network and how these could have affected a potential spatial invasion of SARS-CoV-2. It was beyond our intention to reproduce the spatial invasion of the virus or to assess whether municipalities laying on the most resilient routes were also the most affected ones in terms of cases. However, the reviewer's observation is certainly an interesting and impactful research question that we could explore in future work with genomic surveillance. We rephrased the Limitations Section to better emphasize the possible exploration of such a question in a forthcoming study and added the new analysis and figure in the Supplementary Material Section *Further epidemic impact insights*.

2. *<<Second, while your study already highlights socioeconomic factors, I would encourage further reflection on how territorial and spatial characteristics of the municipalities could contribute to advancing the current model. For example,*

incorporating a spatial heterogeneity analysis that focuses on rural versus urban divisions, physical connectivity, or infrastructural limitations might reveal additional complexities in the mobility network's response to interventions. Understanding how these territorial dimensions interact with socioeconomic factors could offer new avenues for expanding the model and enhancing its applicability to different geographical contexts. This could be particularly valuable when considering public health strategies tailored to specific territorial realities.>>

We thank the reviewer for this insightful question.

The rural/urban divide is kept into account by the variable "urbanization" in our regressions, which is defined as the percentage of the population living in urbanized areas (see **Table 1** in the paper). This variable also proved to be statistically significant across all three regressions. Since we were not clear enough, we brought more attention to this specific variable in Section "*Sociodemographic, epidemiological and mobility network metrics for Chile*" and the importance of considering a measure of the urban-rural divide.

Physical connectivity and infrastructural limitations, instead, are directly captured by our network metrics, which represent typical traffic patterns under a business-as-usual context. Additionally, physical connectivity in terms of geographical proximity is incorporated in the spatial lag model via the proximity matrix W (Equation 4 in the paper). We specified in subsection *"Mobility Data Analysis"* of the main paper that the infrastructural and connectivity aspects are already encoded in the network metrics employed in the regression.

Furthermore, we aimed to include many more socio-economic features that could impact the mobility network. For this reason, we used the IDC (Development Index of Comunas) which is a composite metric that includes several aspects such as health, local economy, and education. The table with the composition of this metric can be found at (reference [39] https://www.uautonoma.cl/noticias/indice-de-desarrollo-comunal-2020-refleja-altos-indices-de-desigualdad-en-el-desarrollo-comunal-de-chile/) and we now reference it in the paper. To ease the reviewer, we share the table here.

DIMENSIÓN	VARIABLE	DESCRIPCIÓN	AÑO DEL DATO
Salud y bienestar social	Servicios básicos	Porcentaje de hogares sin servicios básicos	2017
	Pobreza	Porcentaje de hogares en situación de pobreza	2017
	Agua potable	Porcentaje de cobertura de agua potable comunal	2018
	AVPP	Años de vida potenciales perdidos	2014
Economía y recursos	Dependencia etaria	Dependencia etaria (población inactiva > 65 años que depende de la PEA)	2017
	Ingresos propios permanentes	Ingresos propios permanentes municipales	2018
	Internet	Conexiones a internet fijas por habitante	2018
	Empresas	Número de empresas por habitante	2017
Educación	Simce lenguaje	Puntaje promedio Simce lenguaje octavo básico	2017
	Simce matemáticas	Puntaje promedio Simce matemáticas octavo básico	2017
	Matrículas párvulos	Matrículas educación parvularia respecto a población entre 4 y 5 años	2018
	Matrículas enseñanza media	Matrícula educación media respecto a población entre 14 y 17 años	2018
	PSU	Promedio de porcentaje de alumnos con puntajes PSU sobre 450 puntos en colegios municipales, subvencionados y privados	2018

FUENTE: elaboración propia

Table R1: The composition of the IDC metric. The IDC is based on health, wealth, local economy and resources and education. Health and social wealth are estimated through basic services, poverty index, drinking water, and potential years of life lost. Economy and resources are estimated through the active population, local income, internet access, and local entrepreneurship. Education score is estimated through high school final and partial test scores in mathematics and languages, enrolled students in primary and secondary schools.

Reviewer 4:

1. *<<The regression model includes many covariates but the implementation of a NPI was not one of them. It seems to me that it would be the main driver of a reduction in mobility (that, and the implementation of NPI in the closest neighbors) and unless I missed something, it was not included in the model. I expect that including it as a covariate would lead to major changes in the model interpretation. To illustrate my point, age and gender were found to be significant factors for the change in mobility between the second and first wave. The authors explain this by stating that scarcely populated areas with a higher development index are mostly populated by young males. However, I am not convinced that those factors are drivers of the reduction in mobility. Rather, it could be that those specific regions with younger males were targeted by the NPIs during the second wave but not during the first wave.>>*

We thank the reviewer for raising concerns about the lack of clarity on this point. The NPI should indeed have a major role in determining mobility but, while data on NPIs at municipality level are available, they were not directly included as covariates in the regression models presented in the main manuscript for two main reasons that were not sufficiently discussed. The first reason is that, in both waves, the variance of the variable

modeling the NPI adoption has the same value in almost all comunas. This can be seen in **Figure 1**, where only a tiny fraction of all municipalities in the first wave were under NPIs, while in the second wave, almost the entire country was under restrictions. The lack of heterogeneity makes it incapable of explaining mobility reductions. Secondly, the two intervention periods were characterized by distinct restriction strategies: the first wave was based on a binary classification, while the second wave was based on a tiered system of increasing stringency restrictions by the Chilean government. As a consequence, when considering this variable in the regression models, one needs to perform some arbitrary choices to parametrize and compare the two waves.
For these reasons, we originally chose to exclude this variable from the analysis, but we agree with the reviewer that this should be discussed more clearly. We thus added a paragraph in the Limitations section to clarify this point. Moreover, to bring additional evidence, we conducted an analysis incorporating NPI variables directly into the Spatial-Lag regression models, that we succinctly describe hereafter. The results show that the role of the NPI variables are significant, but minor and do not change the interpretation of all other parameters. For this reason, we plan to keep the analysis without the NPI in the main text (since it is independent of the arbitrary choices made to include this variable) and defer this deeper analysis to the supplementary material, see **Supplementary Table 1-2**.

Here we dive more into the details of the sensitivity analysis. For the first wave 'NPI' is a binary variable, which is 1 for an active NPI during any day of the considered week; or 0 otherwise. For the second wave, NPI is a categorical variable 'Paso', indicating the stage of the 'Paso a Paso' plan for each municipality during that week. This variable takes integer values ranging from 1 (severe restrictions) to 5 (no restrictions), see Pappalardo, L., Cornacchia, G., Navarro, V. *et al.* A dataset to assess mobility changes in Chile following local quarantines. *Sci Data* 10, 6 (2023). https://doi.org/10.1038/s41597-022-01893-3, https://www.nature.com/articles/s41597-022-01893-3.
To ensure consistency between the two waves, we binarized the 'Paso' variable. Municipalities in the first two stages of the 'Paso a Paso' plan are considered under restrictions (i.e., NPI = 1), while those in stages 3 to 5 are classified as having no restrictions. As a result, each week during the two considered waves features a binary variable, 'NPI,' indicating the presence or absence of imposed restrictions in the considered municipality. Once binarized, we tested different aggregation methods of the NPI variable. We defined NPI as a dummy variable assuming value 1 when municipalities experience restrictions for at least 1/2/3 or weeks during the period under study. Finally, we defined a numerical variable for NPI representing the number of weeks under restrictions in the period under study.
In **Supplementary Table 1 and 2** we performed a sensitivity analysis of the Spatial Lag model including all these alternative treatments of the NPI variables. The first column of **Supplementary Table 1 and 2** shows the results already presented in the main text, where information regarding NPIs is not considered. The other columns show the regression coefficients for all covariates obtained using different aggregation procedures for the NPI variable, along with their statistical significance. Although the NPI variable turns out to be always statistically significant, the results are not significantly different from what is already shown in the main text excluding NPIs from the covariates. Some other

covariates might lose statistical significance (e.g. the municipal development index IDC) but all key findings from the main text are still holding (e.g. the strong relevance of baseline network variables and the presence of spatial autocorrelations effects).
As shown in **Supplementary Table 1 and 2** bottom, including the NPI variable does not result in a significant increase in any of the goodness-of-fit metrics considered.

	No NPI	1 week	2 weeks	3 weeks	4 weeks	Continuos
	β	β	β	β	β	β
intercept	-0.24***	-0.24***	-0.24***	-0.25***	-0.24***	-0.24***
age	0.01*	0.01	0.01	0.01	0.01*	0.01
urbanization	0.04***	0.04***	0.04***	0.04***	0.04***	0.04***
gender ratio	-0.01	-0.01	-0.01	-0.01	-0.01	-0.01
dependency	-0.04***	-0.04***	-0.04***	-0.04***	-0.04***	-0.04***
schooling	-0.01	-0.01	-0.01	-0.01	-0.01	-0.01
primary	0.00	0.00	0.00	0.00	0.00	0.00
tertiary	-0.08***	-0.08***	-0.08***	-0.08***	-0.08***	-0.08***
pop density	0.01	0.01	0.01	0.01	0.01	0.01
log(pop)	0.01	0.00	0.00	0.00	0.01	0.00
IDC	-0.03**	-0.03*	-0.03*	-0.03*	-0.03**	-0.02*
New Cases	-0.01**	-0.00	-0.00	-0.01*	-0.01**	-0.00
cl. coeff.	0.02**	0.02**	0.02*	0.03**	0.02**	0.02**
betweenness	-0.01**	-0.01*	-0.01*	-0.01*	-0.01**	-0.01*
in-path-length	0.04***	0.04***	0.04***	0.04***	0.04***	0.04***
out strenght pc	0.03***	0.03***	0.03***	0.03***	0.03***	0.03***
ρ (spatial lag)	0.35***	0.34***	0.33***	0.34***	0.35***	0.33***
NPI		-0.08**	-0.08**	-0.09*		-0.14**
Pseudo R^2	0.53	0.55	0.55	0.55	0.53	0.56
τ_K	0.53***	0.54***	0.54***	0.54***	0.53***	0.54***

Supplementary Table 2: Sensitivity to NPI variable of correlates of mobility change in the first wave. We report the estimated coefficients without the NPI dummy variable (first column), including municipalities that were under NPI at least for one week, two, three, and four weeks, and defining NPI as a continuous variable encoding the number of weeks the municipality spent under NPI (right column). The spatial lag model coefficients, β for covariates and ρ for spatial lag from Eq.5 are reported with the goodness of fit measured by R2 and the Kendall's Tau (τK) coefficient between observed and predicted mobility drops. We report significance as: ***=99.9%, **= 99%, *= 95%. Positive coefficients represent higher mobility.

	No NPI	1 week	2 weeks	3 weeks	4 weeks	Continuos
	β	β	β	β	β	β
intercept	-0.21***	-0.14***	-0.13***	-0.17***	-0.14***	-0.11***
age	0.00	0.00	0.00	0.00	0.00	0.00
urbanization	0.06***	0.06***	0.06***	0.06***	0.06***	0.06***
gender ratio	0.00	-0.00	-0.00	9.23	0.00	-0.00
dependency	-0.07***	-0.07***	-0.07***	-0.07***	-0.07***	-0.07***
schooling	0.00	0.01	0.01	0.01	0.01	0.01
primary	-0.03*	-0.03*	-0.03**	-0.03*	-0.03**	-0.03**
tertiary	-0.11***	-0.12***	-0.12***	-0.12***	-0.12***	-0.12***
pop density	0.02*	0.02*	0.02*	0.02*	0.02*	0.02*
log(pop)	-0.01	-0.01	-0.01	-0.01	-0.00	-0.00
IDC	-0.04*	-0.04**	-0.04**	-0.04**	-0.04**	-0.04**
new cases	-0.00	-0.00	-0.00	-0.00	0.00	0.00
deaths	0.00	0.00	0.00	0.00	0.00	0.00
test rate	0.00	0.00	0.00	-0.00	-0.00	-0.00
clustering	-0.00	-0.00	-0.00	-0.00	-0.00	-0.00
betweenness	-0.01	-0.01	-0.01	-0.01	-0.01	-0.01
in-path-length	0.10***	0.10***	0.10***	0.10***	0.10***	0.10***
out-strength pc	0.01*	0.02*	0.02**	0.01*	0.01*	0.02*
ρ (spatial lag)	0.19***	0.19***	0.18***	0.18***	0.17**	0.17**
NPI		-0.07*	-0.08**	-0.04*	-0.08***	-0.11***
Pseudo R^2	0.58	0.58	0.59	0.59	0.60	0.59
τ_K	0.38***	0.39***	0.4***	0.39***	0.4***	0.4***

Supplementary Table 2: Sensitivity to NPI variable of correlates of mobility change in the second wave. We report the estimated coefficients without the NPI dummy variable (first column), including municipalities that were under NPI at least for one week, two, three and four weeks and defining NPI as a continuous variable encoding the number of weeks the municipality spent under NPI (right column). The spatial lag model coefficients, β for covariates and ρ for spatial lag from Eq.5 are reported with the goodness of fit measured by R2 and the Kendall's Tau (τK) coefficient between observed and predicted mobility drops. We report significance as: ***=99.9%, **= 99%, *= 95%. Positive coefficients represent higher mobility.

We reported the sensitivity analysis and the relative description of the alternative aggregations in the Supplementary Information Section *Sensitivity to NPI dummy*

variable with the relative Tables and referenced them in the Discussion Section of the main text.

2. *<<The map in Fig 1B is hard to read and would be better with four distinctive colors.>>*

We agree that the colors were not clear enough, we followed the reviewer's suggestion and discretized the colorbar using 4 distinctive colors and rephrased the caption to better explain the legend of the heatmap. See below the new **Figure 1** and the new caption.

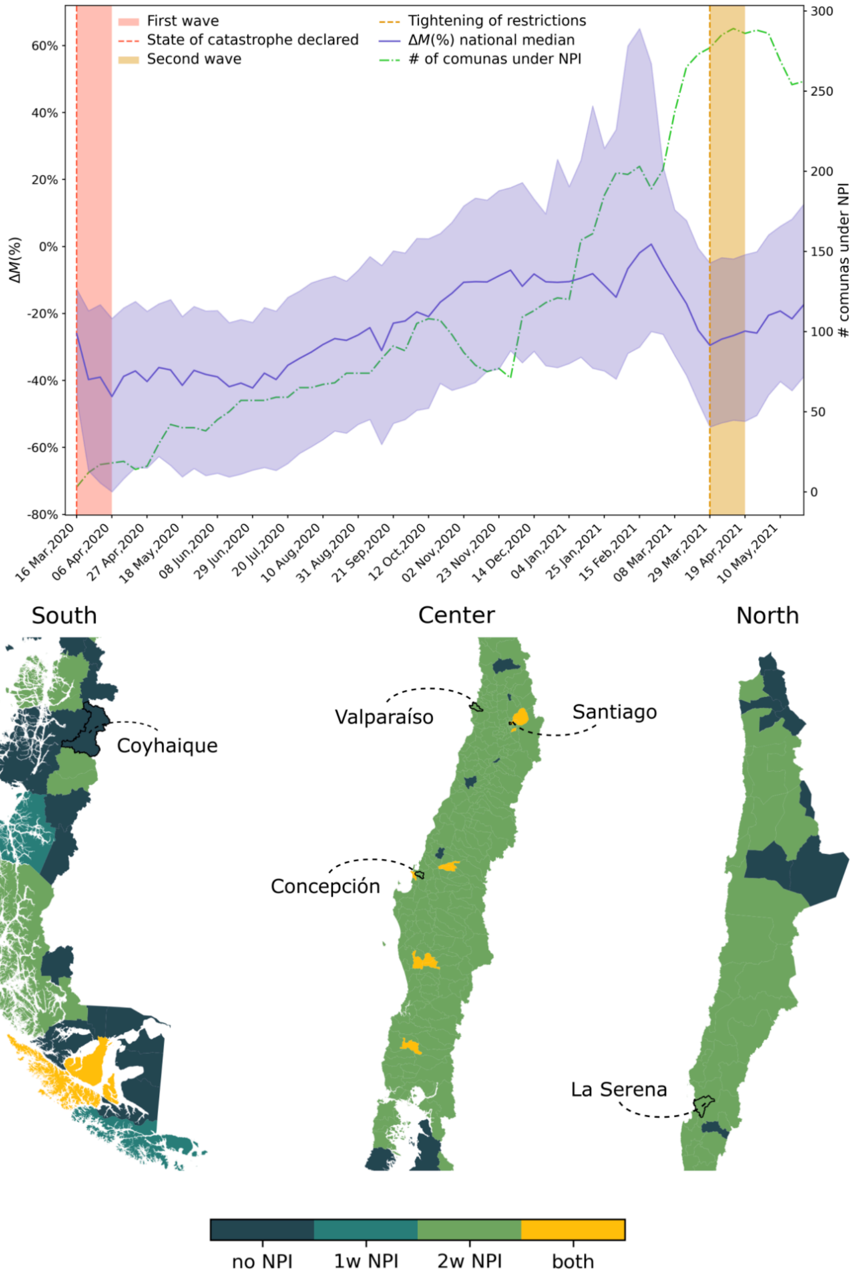

Figure 1. The first and second wave in Chile. On top: weekly median mobility change with respect to the baseline (purple solid line) and respective 95% interval across municipalities (purple shaded area). The dashed-dotted green curve represents the number of municipalities

under restrictions, while the vertical areas correspond to the first (red) and second (yellow) waves. On the bottom, the map of three macro-regions of Chile shows municipalities under restrictions. The color code represents municipalities according to the NPIs they experienced. We make the distinction for adoption only in the first wave (light blue, 1w NPI), only in the second wave (green 2w NPI), in both waves (yellow, both) and neither (dark blue, no NPI).

Editor's notes:

1. *<< [...] it's not clear how interventions were included in the regression model. Are they exclusively captured by in the mobility data and stratification of the analysis between intervention periods? If so, I might suggest a unified analysis with indicator variables associated with interventions (or continuous/ordinal).>>*

See answer to reviewer comment R4.1

2. *<<Second, the authors state that the network structures were strikingly similar. While I agree there are strong similarities, looking at the scatter plots of the Pijs there are many which have changed by 2+ orders of magnitude. I agree with the reviewers that more work is needed to articulate which aspects of the networks were retained and which were changed. I think you have this information in the regression analysis and do discuss it a bit, but I believe the work would be far strengthened with a deeper dive into differences/similarities in the networks.>>*

We thank the editor for this constructive suggestion. Accordingly, we performed supplementary analyses to highlight how the main features of the network changed between the baseline period and the second wave period.
In the newly added **Supplementary Figure 6** we show the distribution of the outstrength and the outstrength per-capita, encoding the total outbound trips and total outbound trips per-capita for all municipalities in Chile in the three periods. From this comparison, we see that the two intervention periods exhibit very similar heterogeneous distributions of outbound trips, especially compared to the baseline period.

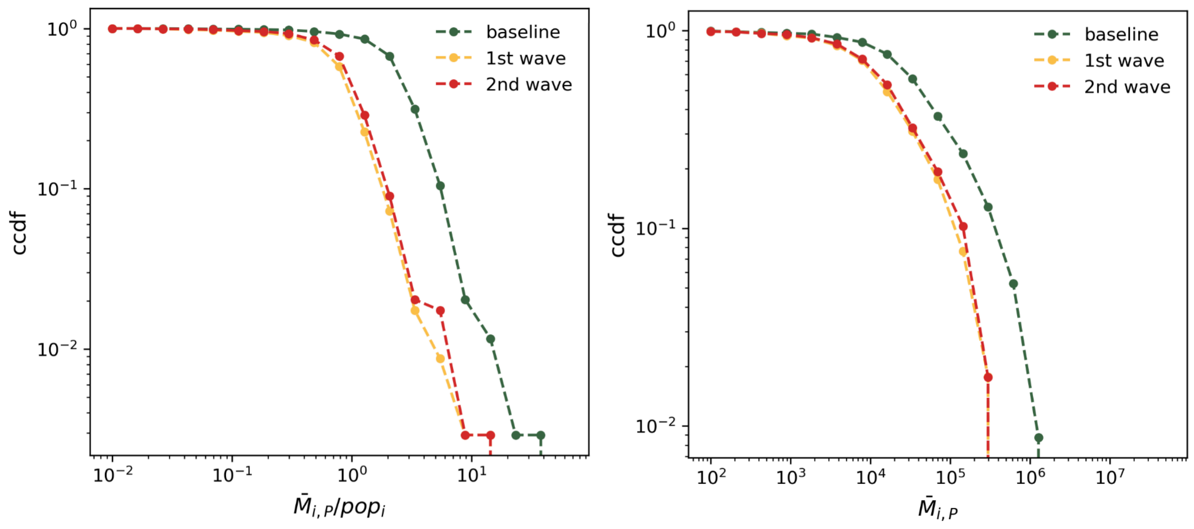

Supplementary Figure 6: Distribution of mobility network outstrength of baseline, first and second wave period. Log-log plot of the cumulative complementary density function of mobility network outstrength per capita on the left, and outstrength on the right.

To deepen our analysis of the network structural changes, we focus on three main cities consistently to response 3.1 which we addressed to Reviewer 3. Here we compare the resilience of the main outbound routes between the baseline and second wave periods in relation to the clustering coefficient and in-path length of destination municipalities. As shown in **Supplementary Figure 7**, circles that deviate the most from the diagonal, hence the least resilient routes, are typically headed towards less clustered nodes with respect to most resilient routes. This can be interpreted as mobility clusters not altering their behavior during intervention periods due to economic activity dependence between neighboring towns. For central towns like Santiago and Valparaiso, the least resilient routes are also headed towards more peripheral areas, characterized by brighter colors in the plots, encoding higher in-path length in the network. The effect is less clear in Concepción as few routes deviate sensibly from the diagonal. This confirms our results obtained in the regression model suggesting that peripherality correlates positively and significantly with higher mobility with respect to the baseline period.

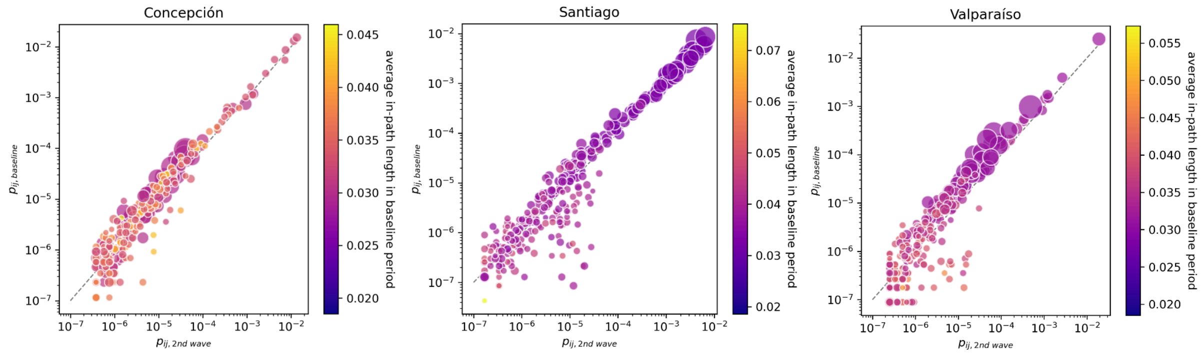

Supplementary Figure 7: Insight on routes mobility resilience related to clustering and peripherality of destination nodes. On the y-axis the traveling probability from three major municipalities *i* to *j* in the baseline period vs the second wave period on the x-axis. Plot title

encodes the origin *i*. Circles correspond to destination municipalities. Circle thickness encodes the clustering coefficient of municipalities, with thinner circles for less clustered nodes and thicker circles for more clustered nodes. Color code encodes the average in-path length of destination municipalities, with darker colors for more peripheral nodes, and brighter colors for more central nodes.

To dive more into the network features correlated with mobility routes resilience, we extended our analysis to the relation with outstrength per-capita. In **Supplementary Figure 8**, we show the same scatter plot of Supplementary Figure 10, this time with circle thickness encoding outstrength per-capita of destinations in the baseline period. In all the plots we can appreciate how the least resilient routes are generally headed towards destinations with low outstrength per-capita. This confirms the results of the regression models, which highlighted the positive and significant correlation of outstrength per-capita correlation with higher mobility during the second wave with respect to the baseline.

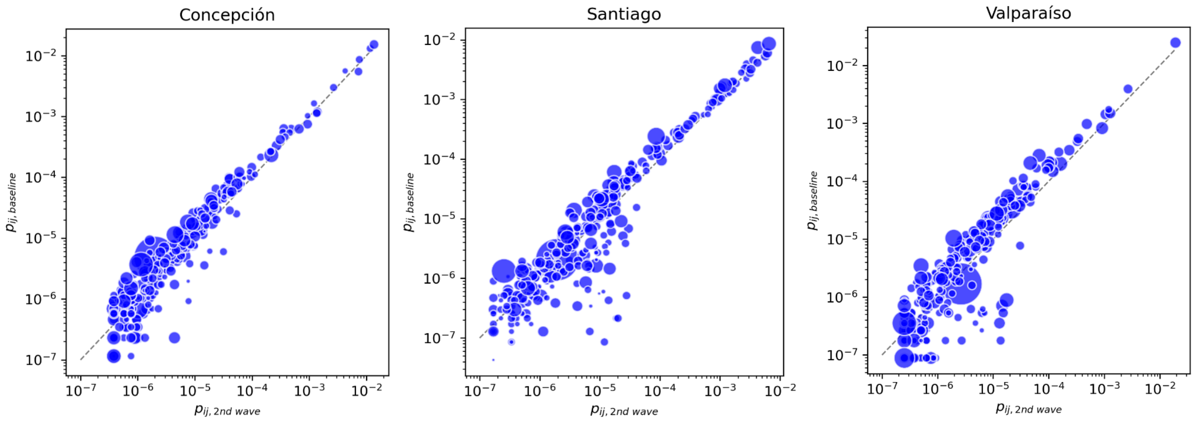

Supplementary Figure 8: Insight on routes mobility resilience related to out-strength per-capita of destination nodes. On the y-axis the traveling probability from three major municipalities *i* to *j* in the baseline period vs the second wave period on the x-axis. Plot title encodes the origin *i*. Circles correspond to destination municipalities. Circle thickness encodes the outstrength per-capita measured in the baseline period, with thinner circles for municipalities with higher trips per-capita and thicker circles for municipalities with higher trips per-capita in the baseline period.

We thank the Editor for suggesting this analysis that definitely enriches the quality of the manuscript. These results were added to the Supplementary Material Section *Further insights on the network features associated with network resilience* and referenced them in the main text Results Section.

3. *<<I agree with the reviewers that contextualizing the mobility changes in the context of number of cases would help us understand the impact of the interventions. I realize that measuring this impact is not the focus of the paper (which is fine) but again I think you have this information coming from the regression or could easily determine it with a slightly modified regression.>>*

See answer to reviewer comment R3.1

4. *<<In the first regression model I see where absolute mobility enters into the analysis, i.e., via the strengths in the mobility network, but in the second analysis I agree that more motivation is needed for why relative change is more meaningful than absolute change. I understand why relative change is important, so I'm not suggesting abandoning that analysis, instead I think you could compare/contrast the absolute change (which will impact transmission).>>*

We thank the editor for the suggestion. In the main text, we focused on the relative change of mobility in order to discard the population bias which could alter the comparison across comunas due to high heterogeneity in municipality size, see **in Figure R1** below.

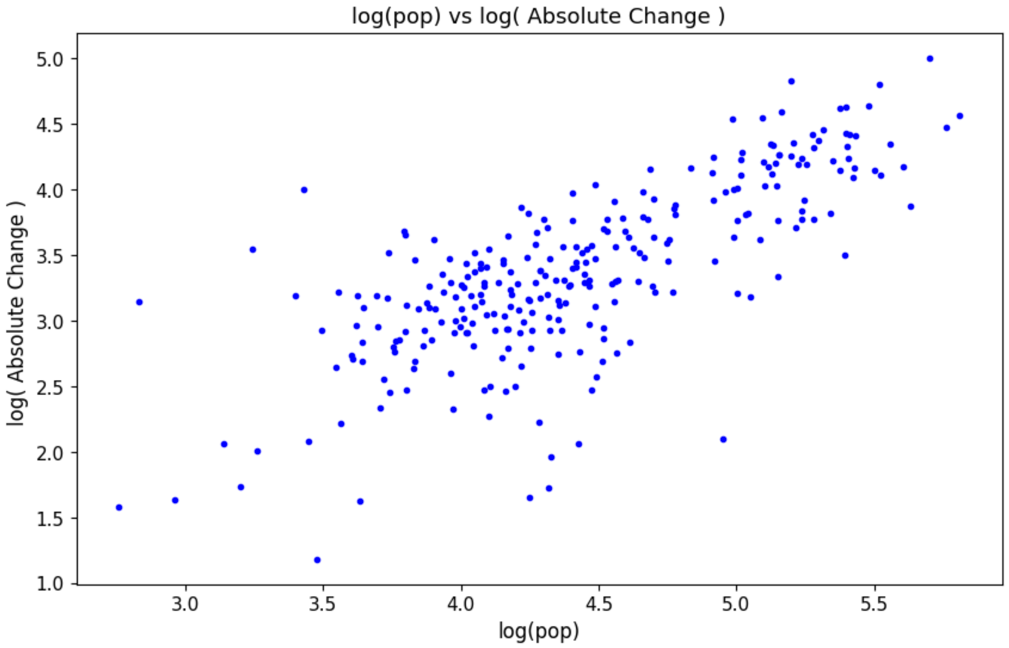

Figure R1: Comparison of population size and absolute change between the first and second wave at municipality scale. On the y-axis the absolute outflow change between the second and first wave of Chilean municipalities, on the x-axis the logarithm of the population size of municipalities. 15 municipalities exhibited a negative absolute change and were removed from the scatter plot to allow for a log-scale comparison for the sake of visualization.

The distribution of mobility absolute change exhibits some important outliers, as shown in **Figure R2** below, most municipalities increased outbound trips between 0 and 20000 units, while some municipalities exhibited sensibly higher trips increase, topping at 100000 units. Outliers like this could greatly affect the estimates of coefficients in regression models. On the other hand, the distribution of relative change exhibits less skewness and a more homogeneous distribution around a 20% average, see **Figure R3.**

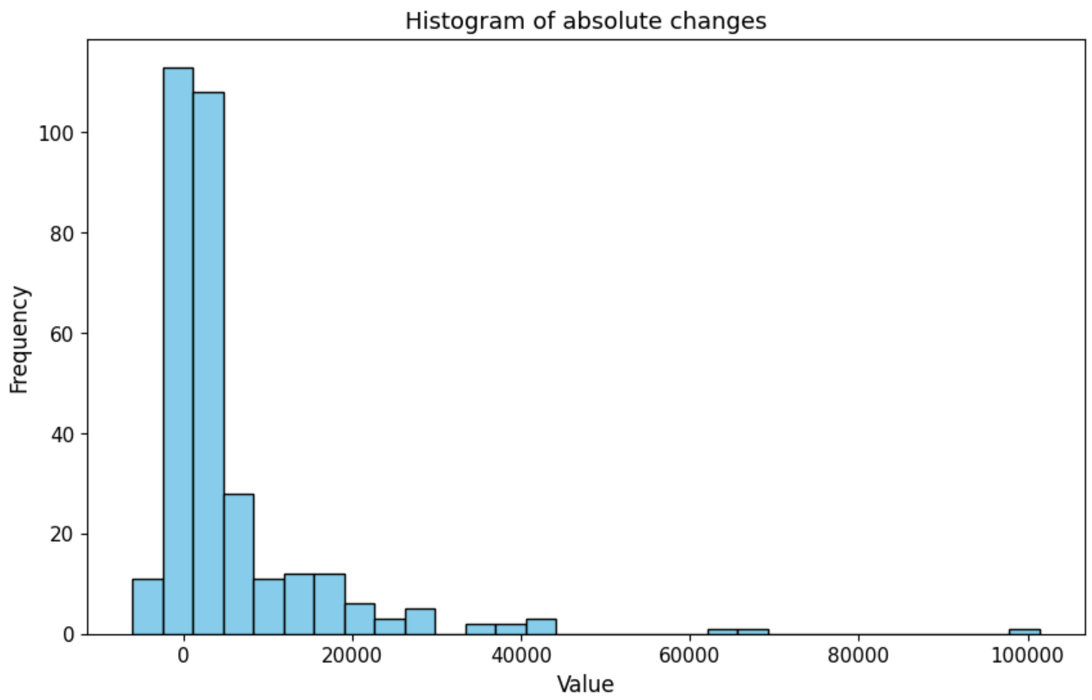

Figure R2: Distribution of mobility absolute change between first and second wave of Chilean municipalities. Mobility is measured in terms of outflows.

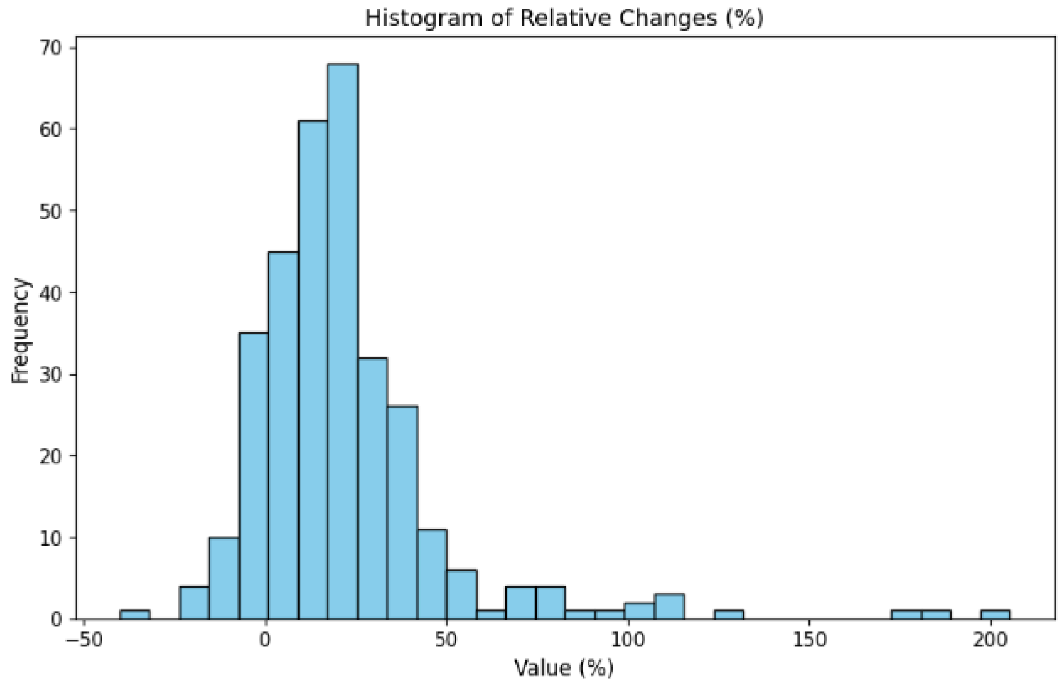

Figure R3: Distribution of mobility relative change between first and second wave of Chilean municipalities. Mobility is measured in terms of outflows.

Following the editor's suggestion, we repeated the regression using the absolute change of mobility as target variable. As shown in **Table R2**, some of the coefficients are significant in explaining both relative and absolute mobility change, like urbanization,

primary sector, and development index (IDC). Median age and gender ratio significantly explain only relative change. Other coefficients are significant in explaining exclusively absolute changes, like schooling, betweenness centrality, and population density. The latter presumably absorbs the effect of the population observed in **Figure R1** above. However, we refrain from diving into more detailed interpretation of the coefficients explaining absolute changes since few outliers, driven by population size, typically correlated to urbanization levels, schooling level, tertiary sector, and economic development are probably impacting coefficient estimates.

	β **relative**	[0.025	0.975]	β **absolute**	[0.025	0.975]
intercept	-0.0	-0.079	0.079	-0.0	-0.062	0.062
age	-0.21^{***}	-0.324	-0.103	-0.05	-0.133	0.042
urbanization	-0.17^{*}	-0.323	-0.010	-0.27^{***}	-0.397	-0.150
gender ratio	0.24^{**}	0.067	0.404	0.04	-0.088	0.177
dependency	-0.05	-0.222	0.116	-0.09	-0.225	0.041
schooling	0.12	-0.068	0.306	0.28^{***}	0.133	0.428
primary	-0.31^{**}	-0.497	-0.116	-0.24^{**}	-0.393	-0.093
tertiary	0.24^{*}	0.015	0.456	-0.28^{***}	-0.458	-0.111
pop density	0.12	-0.015	0.251	0.25^{***}	0.148	0.357
log(pop)	-0.53^{***}	-0.719	-0.346	-0.01	-0.152	0.141
IDC	0.31^{**}	0.095	0.523	0.35^{***}	0.184	0.520
clustering	-0.4^{***}	-0.565	-0.239	0.2^{**}	0.072	0.329
betweenness	0.06	-0.040	0.162	0.24^{***}	0.160	0.319
in-path-length	0.18^{***}	0.085	0.266	0.02	-0.049	0.094
out-strength oc	-0.25^{***}	-0.350	-0.148	-0.02	-0.096	0.062
cases increment	-0.01	-0.097	0.076	0.01	-0.060	0.077

Table R2: Correlates of absolute mobility change across the two waves in Chile. Linear regression coefficients β and respective 95% confidence interval for the model of Eq. (5). Positive coefficients represent higher mobility flows associated with higher covariates.

5. *<<In terms of additional historical context, I leave it up to the authors to decide whether that is needed.>>*

We have addressed the reviewer's concern, see response to R2.1.
